# The impact of age and 24‐h blood pressure on arterial health in acute ischemic stroke patients: The Norwegian stroke in the young study

**DOI:** 10.1111/jch.14361

**Published:** 2021-09-07

**Authors:** Sahrai Saeed, Ulrike Waje‐Andreassen, Halvor Naess, Annette Fromm, Peter M. Nilsson

**Affiliations:** ^1^ Department of Heart Disease Haukeland University Hospital Bergen Norway; ^2^ Department of Neurology Haukeland University Hospital Bergen Norway; ^3^ Department of Clinical Sciences Lund University Skane University Hospital Malmö Sweden

**Keywords:** age, ambulatory blood pressure, arterial stiffness, carotid intima‐media thickness, hypertension, ischemic stroke, pulse pressure, pulse wave velocity

## Abstract

The impact of age and 24‐h ambulatory blood pressure (ABPM) on arterial stiffness and carotid intima‐media thickness (cIMT) in ischemic stroke patients younger than 60 years of age is poorly explored. A total of 385 acute ischemic stroke patients (aged 49.6±9.7 years, 68% men) were prospectively included and grouped in younger (15–44 years, *n* = 93) and middle‐aged (45–60 years, *n* = 292). Arterial stiffness was measured by carotid‐femoral pulse wave velocity (PWV), and cIMT by carotid ultrasound. 24‐h ABPM was recorded. The middle‐aged stroke patients had higher prevalence of smoking, hypertension, diabetes mellitus, metabolic syndrome and hypercholesterolemia, and had higher PWV and cIMT (all *p* < .05). In multivariable linear regression analyses adjusted for sex, BMI, smoking, diabetes mellitus, total cholesterol, high‐density lipoprotein cholesterol, triglycerides, eGFR, systolic BP and concomitant antihypertensive treatment, 1SD (4.4 years) higher age was associated with higher PWV (β = 0.44,R^2 ^= 0.46, *p* < .001) in the younger group, and with higher mean cIMT (β = 0.16, R^2 ^= 0.21, *p* = .01) in the middle‐aged group. In the middle‐aged group, 24‐h pulse pressure had a significant association with PWV (β = 0.18, R^2 ^= 0.19, *p* = .009), while the association with cIMT was attenuated (β = 0.13, R^2 ^= 0.16, *p* = .065). 24‐h diastolic BP was associated with higher cIMT in the middle‐aged group (β = 0.24, *p* < .001, R^2 ^= 0.23), but not with PWV in either age groups. Among ischemic stroke patients < 60 years, higher age was associated with increased arterial stiffness for patients up to age 44 years, and with cIMT in middle‐aged patients. 24‐h pulse pressure was associated with arterial stiffness, and 24‐h diastolic BP was associated with cIMT only in middle‐aged patients.

## 1 INTRODUCTION

Aging is one of the most powerful risk factors for cardiovascular (CV) disease, particularly ischemic stroke, and the risk increases exponentially.^1^ Although the majority of stroke patients are elderly, up to one‐third of first‐ever strokes occur in patients younger than 65 years.^2^, ^3^ Compared to older stroke patients, mortality rates in younger patients are low, but the rates of morbidity are substantially high,^4^ particularly the risk of recurrent vascular events in long‐term stroke survivors.^5^ Etiology and risk factors of ischemic stroke differ significantly in younger and older patients.^6^ However, some risk factors may be shared, that is, hypertension being one of the most important modifiable risk factors for ischemic stroke at any age. Hypertension is also frequently associated with increased arterial stiffness and altered left ventricular (LV) geometry and function contributing to the high risk for recurrent CV events in these patients.^7^ However, the relative impact of a risk factor may change during aging, and it is therefore difficult to compare the strength of a particular risk factor in younger versus older patients.^8^, ^9^ Thus, a deeper understanding of the CV risk profile in different age categories of ischemic stroke patients may contribute to develop a more personalized approach to secondary prevention. 24‐h ambulatory blood pressure (ABPM) is a strong predictor of CV morbidity and mortality and closely related to target organ damage compared to clinic BP.^10^ In the present study, we aimed to assess the interaction between age, 24‐h ABPM components and traditional CV risk factors with the indices of subclinical arterial damage (carotid‐femoral pulse wave velocity [PWV], and carotid intimal‐media thickness [cIMT]) in young and middle‐aged patients with a previous acute ischemic stroke.

## 2 METHODS

### 2.1 Study population

The general study design, inclusion and exclusion criteria of the Norwegian Stroke in the Young Study (NOR‐SYS) have been previously published in detail.^11^ Briefly, in the NOR‐SYS registry a total of 385 patients aged 15–60 years with a documented ischemic stroke were included between September 2010 and August 2015 as key persons of a 3‐generation research program. After discharge, patients were referred to the Department of Heart Disease, Haukeland University Hospital, Bergen, Norway, for 24‐h ABPM recording and assessment of arterial stiffness.^7^ As previously described, serious sequelae after the index stroke event, death and repeated failure to attend the out‐patient visits were the main reasons for drop‐out during follow‐up.^7^ The study was approved by the Regional Committee for Medical Research Ethics of Western Norway and conducted in accordance with the Declaration of Helsinki. All patients or their legal representatives signed a written informed consent.

### 2.2 Assessment of cardiovascular risk factors

Clinic BP was measured according to current European hypertension guidelines after approximately 5 min rest in the sitting position.^12^ Pulse pressure (PP) was calculated as systolic BP minus diastolic BP. A 24‐h ABPM recording was performed using a Diasys Integra II device (Novacor, Cedex, France) approximately 3 months after the index stroke event as previously described.^7^ The device was pre‐set to automatically measure heart rate and BP at 30 min intervals during night‐time and 15 min intervals during day‐time, providing a total of 78 measurements over 24‐h. Day‐time was defined as the interval between 07:00 and 23:00 h and night‐time between 23:00 and 07:00 h. In the case of < 70% valid BP recordings, the ambulatory BP recording was repeated. Hypertension was defined as a history of hypertension, use of antihypertensive medications, persistently elevated BP during hospitalization, or elevated clinic BP (≥140/90 mm Hg) or 24‐h BP (≥130/80 mm Hg) at the 3‐months follow‐up visit.^12^ Uncontrolled treated hypertension was defined as elevated 24‐h ambulatory BP (≥130/80 mm Hg) in treated hypertensive patients.

### 2.3 Assessment of arterial function and structure

The method for assessment of arterial function and structure has been published elsewhere in details.^7^ In brief, cIMT was measured by ultrasound at admission for the index stroke, and arterial stiffness by carotid‐femoral PWV using a Sphygmocor device (AtCor Medical, Sydney, West Ryde, Australia).^13^ Target arterial damage was defined as mean common cIMT > 0.9 mm, presence of high for age PWV or PWV > 10 m/s.^12^ High‐for‐age PWV was defined as PWV higher than age‐adjusted normal range values based upon the Anglo‐Cardiff Collaborative Trial.^14^ Carotid plaque was defined as focal cIMT > 1.5 mm at the site of common carotid artery.^7^

Venous blood samples were drawn for analysis of fasting serum lipids and glucose. Diabetes mellitus was defined as history of diabetes, use of anti‐diabetic treatment or fasting blood glucose ≥7 mmol/L. Metabolic syndrome was defined according to the modified American Heart Association/National Heart, Lung, and Blood Institute criteria.^15^ Estimated glomerular filtration rate (eGFR [ml/min/1.73 m^2^]) was determined from serum creatinine by the Chronic Kidney Disease Epidemiology Collaboration equation (CKD‐EPI). Body mass index (BMI) > 30 kg/m^2^ was considered as obesity.

### 2.4 Statistical analysis

Analyses were performed using the IBM SPSS statistical program version 26 (IBM, Armonk, New York, USA). The data is presented as mean ± standard deviation for continuous variables and as percentages for categorical variables. Patients were grouped as young (15–44 years) or middle‐aged (45–60 years), and inter‐group comparison was done by independent student *t* test and chi‐square test as appropriate. The association of age with traditional CV risk factors and arterial health parameters (PWV and cIMT) was tested in univariate and multivariate linear regression analyses in both age groups separately, and reported as standardized β‐coefficients and *p*‐values. Multivariate models were adjusted for sex, BMI, smoking, diabetes mellitus, hypertension, total cholesterol, HDL cholesterol, triglycerides, eGFR, systolic BP and concomitant antihypertensive treatment. A *p* value < 0.05 was taken as statistical significance in all analyses.

## 3 RESULTS

### 3.1 Patient characteristics

**FIGURE 1:**
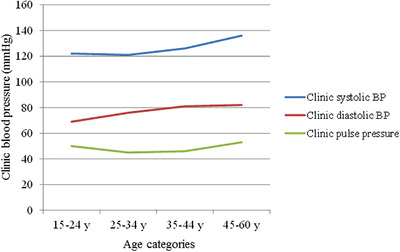
Trends in clinic systolic blood pressure, diastolic blood pressure and pulse pressure across the age categories

The study population included 385 patients (mean age 49.6±9.7 years, 68% men). Ninety‐three (24%) patients were younger and 292 (76%) were middle‐aged. The middle‐aged group had higher prevalence of traditional CV risk factors including smoking, hypertension, metabolic syndrome, diabetes mellitus and hypercholesterolemia compared with the younger group (all *p* < 0.05) (Table 1). Similarly, eGFR, fasting blood glucose and HbA_1c_ were also significantly higher in middle‐aged patients. BMI did not differ between the groups. Although clinic and 24‐h BP were significantly higher in the middle‐aged group, the prevalence of uncontrolled hypertension was comparable in both groups (Table 1, 2). Systolic BP increased on average by 5 mm Hg from age 15–24 years to 35–44 years in the younger age group, but this increase was further doubled in the middle‐aged group (45–60 years) (Figure 1). Diastolic BP tended to increase across the younger age categories, but did not increase further in middle‐aged patients, leading to widening of PP (Figure 1).

**TABLE 1:** Baseline characteristics of the participants according to age categories

	Young (15–44 years) *N* = 93	Middle‐aged (45–60 years) *N* = 292	*p*
Age (years)	35±8	54±4	<.001
Men (%)	54	73	.001
Current smokers (%)	40	58	.003
Body mass index (kg/m^2^)	26.9±6.3	27.4±4.8	.424
Waist circumference (cm)	91±17	98±13	<.001
Obesity (%)	26	27	.795
Hypertension (%)	48	77	<.001
Antihypertensive treatment (%)	37	63	<.001
Uncontrolled treated hypertension (%)	56	55	.932
Diabetes (%)	7	17	.010
Metabolic syndrome^a^ (%)	23	39	.016
Hypercholesterolemia (%)	50	63	.018
History of statin use before admission (%)	5	18	.003
Total cholesterol (mmol/L)	5.2±1.3	5.4±1.2	.051
LDL cholesterol (mmol/L)	3.3±1.1	3.6±1.1	.017
HDL cholesterol (mmol/L)	1.5±0.6	1.3±0.5	.011
Triglycerides (mmol/L)	1.5±0.9	1.7±1.1	.018
Fasting blood glucose (mmol/L)	5.3±1.1	6.1±2.1	<.001
HbA_1c_ (%)	5.3±0.5	6.0±1.1	<.001
eGFR (ml/min/1.73 m^2^)	107±17	92±16	<.001

*Abbreviations*: HbA1c, glycosylated hemoglobin; HDL, high‐density lipoprotein; LDL, low‐density lipoprotein.^.^

^a^Defined according to the 2005 modified American Heart Association/National Heart, Lung, and Blood Institute criteria.^15^

**TABLE 2:** Clinic and 24‐h ambulatory blood pressure measurements and arterial stiffness indices according to age categories

	Young (15–44 years) *N* = 93	Middle‐aged (15–60 years) *N* = 292	*p*
Clinic systolic BP (mm Hg)	124±17	136±18	<.001
Clinic diastolic BP (mm Hg)	78±11	83±10	<.001
Clinic pulse pressure (mm Hg)	46±10	53±12	<.001
24‐h systolic BP (mm Hg)	116±11	121±15	.003
24‐h diastolic BP (mm Hg)	76±8	79±9	.017
24‐h pulse pressure (mm Hg)	40±7	42±10	.046
Daytime systolic BP (mm Hg)	119±11	124±15	.001
Daytime diastolic BP (mm Hg)	78±8	81±9	.014
Night‐time systolic BP (mm Hg)	105±13	109±16	.049
Night‐time diastolic BP (mm Hg)	68±8	72±10	.001
Elevated 24‐h BP^a^ (%)	26	46	.002
Night‐time dipping (%)	12±7	11±7	.792
Non‐dipping BP pattern (%)	34	38	.565
Mean carotid IMT (mm)	0.64±0.14	0.90±0.30	<.001
IMT ≥0.9 mm (%)	5	40	<0.001
Carotid plaque (%)	1	13	.001
Pulse wave velocity (m/s)	6.4±1.1	8.3±1.9	<.001
Pulse wave velocity ≥10 m/s (%)	0	16	<.001
High for age PWV^b^ (%)	4	20	<.001

*Abbreviations*: BP, blood pressure; IMT, intima‐media thickness; PWV, pulse wave velocity.

^a^Defined as ≥130/80 mm Hg.

^b^High for age PWV was defined as PWV higher than age‐adjusted normal range values based upon the Anglo‐Cardiff Collaborative Trial.^14.^

### 3.2 Association of age with arterial stiffness and carotid intima‐media thickness (cIMT)

**FIGURE 2:**
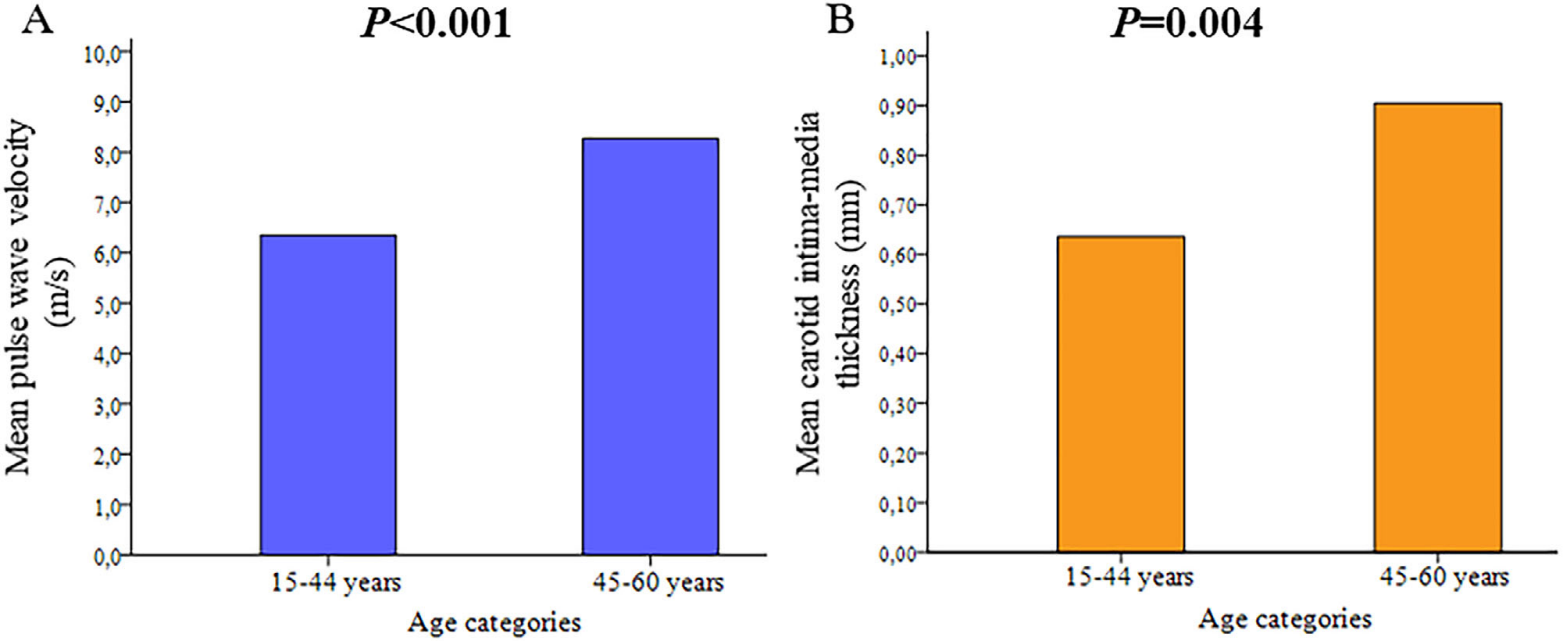
Mean pulse wave velocity (**A**) and mean carotid intima‐media thickness (**B**) in young (15–44 years) and middle‐aged (45–60 years) ischemic stroke patients

Both mean PWV and cIMT were higher in the middle‐aged group than in the younger group (Table 2, Figure 2). Increased cIMT (≥0.9 mm) was found in 40% of middle‐aged patients versus 5% in younger patients (Table 2). All patients in the younger group had a PWV < 10 m/s; among these only 4% had high‐for‐age values. By contrast, in middle‐aged patients PWV > 10 m/s was found in 16% and high‐for‐age PWV in 20% (Table 2). In univariate linear regression analyses, 1SD (4.4 years) higher age was strongly associated with PWV and mean cIMT in the younger group, but was poorly associated in the middle‐aged group (Table 3). In a multivariable linear regression analysis adjusted for sex, BMI, smoking status, diabetes mellitus, hypertension, total cholesterol, HDL cholesterol, triglycerides, eGFR, systolic BP and concomitant antihypertensive treatment, 1SD higher age was associated with significantly higher PWV (β = 0.44, R^2 ^= 0.46, *p* < .001) in the younger group, and with higher mean cIMT (β = 0.16, R^2 ^= 0.21, *p* = .01) in the middle‐aged group (Table 4).

**TABLE 3:** The impact of one Standard Deviation (SD) (4.4 years) higher age on blood pressure and arterial hemodynamics in univariate linear regression analyses

Variable	Age categories			
	Young (15–44 years)		Middle‐aged (45–60 years)	
	β	*p*	β	*p*
Clinic pulse pressure (mm Hg)	−0.10	.363	0.04	.521
24‐h systolic BP (mm Hg)	0.03	.789	0.09	.173
24‐h diastolic BP (mm Hg)	0.35	.002	0.06	.386
24‐h pulse pressure (mm Hg)	−0.34	.002	0.08	.203
Pulse wave velocity (m/s)	0.45	<.001	0.14	.022
Mean carotid IMT (mm)	0.37	<.001	0.19	.002

*Abbreviations*: BP, blood pressure; IMT, intima‐media thickness.

**TABLE 4:** Multivariable linear regression models showing the impact of one Standard Deviation (SD) higher age, 24‐h pulse pressure and diastolic blood pressure on arterial stiffness indices in young and middle‐aged groups

	Pulse wave velocity (m/s)		Carotid IMT (mm)	
	15–44 years	45–60 years	15–44 years	45–60 years
β for 1 SD (4.4 years) higher age	0.44	0.09	0.12	0.16
*p*	<.001	.112	.322	.010
R^2^	0.46	0.24	0.42	0.21
β for 1 mm Hg increase in 24‐h PP	NA	0.18	NA	0.13
*p*	—	.009	—	.065
R^2^	—	0.19	—	0.16
β for 1 mm Hg increase in 24‐h DBP	0.20	0.02	NA	0.24
*p*	.194	.844	—	<.001
R^2^	0.34	0.22	—	0.23

Adjusted for sex, body mass index, smoking, diabetes mellitus, total cholesterol, HDL cholesterol, triglycerides, estimated glomerular filtrate rate, systolic blood pressure and antihypertensive treatment.

*Abbreviations*: DBP, diastolic blood pressure; IMT, intima‐media thickness; PP, pulse pressure; NA, not applicable due non‐significant association in univariate models.

### 3.3 Association of 24‐h ambulatory BP with arterial stiffness and carotid intima‐media thickness

#### 3.3.1 24‐h pulse pressure (PP)

In univariate linear regression models, 24‐h PP was associated with PWV (β = 0.29, *p* < .001) and cIMT (β = 0.20, *p* = .002) in middle‐aged patients, but not in younger patients (β = ‐0.09, *p* = .43 for PWV, and β = ‐0.02, *p* = .89 for cIMT). In multivariable linear regression models adjusted for sex, BMI, smoking status, diabetes mellitus, hypertension, total cholesterol, HDL cholesterol, triglycerides, eGFR and concomitant antihypertensive treatment, 24‐h PP had a significant association with PWV in middle‐aged patients, but the association with cIMT was attenuated in this group (Table 4).

#### 3.3.2 24‐h diastolic blood pressure

In univariate linear regression models 24‐h diastolic BP was associated with PWV both in the younger group (β = 0.40, *p* < .001) and the middle‐aged group (β = 0.20, *p* = .002), but the association with cIMT was significant only in the middle‐aged group (β = 0.29, *p* < .001), not in the younger group (β = 0.12, *p* = .292). In multivariable‐adjusted models, 24‐h diastolic BP was not associated with PWV in either group, but retained a significant association with cIMT in middle‐aged patients (Table 4).

## 4 DISCUSSION

The present observational study of post‐stroke patients provides several important findings. *First*, a progressive increase in arterial stiffness indices was observed in increasing age categories. *Second*, middle‐aged patients had significantly higher burden of concomitant comorbidities compared to younger patients. *Third*, age was more closely correlated with PWV in younger patients, but with cIMT in middle‐aged patients. Finally, 24‐h PP was a determinant of arterial stiffness in middle‐aged patients, but not in younger patients.

### 4.1 The categorization of age

Only a generation ago, people in their 60's would be considered relatively old. However, with better health care and increasing life expectancy, the concept of ageing has considerably changed over the past 50 years. Although there is no universal definition for “young stroke” patients, the term commonly refers to as patients aged 50 years or less.^1^, ^6^ In the large multicenter European Stroke in Young Fabry Patients (SIFAP) study, 18–55 years was defined as young.^16^ Furthermore, most recently in a study by Olesen and coworkers patients < 61 years of age were defined as non‐elderly.^17^ Thus, in view of these considerations, and given the relatively high life expectancy in Norway, our definition of young as below 45 years and middle‐age as 45–60 years seems reasonable.

### 4.2 The impact of age and 24‐h BP on arterial stiffness and cIMT

In stroke patients < 60 years, arterial stiffness has been less studied. A few smaller studies in older stroke patients have demonstrated the association of increased arterial stiffness with impaired prognosis.^18^ Under normal circumstances, human arteries age slowly with increasing chronological age due to structural changes in the arterial wall like fragmentation and degeneration of elastin, increase in collagen and IMT, and cross‐linking of collagen fibers.^19^, ^20^ This is reflected by an exponential increase in PWV and decrease in aortic strain/elasticity.^21^ However, in the presence of cardio‐metabolic risk factors such as obesity, diabetes mellitus and hypertension, arterial aging is accelerated, and the loss of aortic compliance is nearly complete by the age of 50 years.^19^, ^20^, ^22^, ^23^, ^24^ These changes result in loss of buffer function of the aorta. The reflected pulse waves will return to the proximal aorta early in the systole leading to systolic hypertension and cause widening of PP. The CV risk feature after 50 years of age is mainly dominated by increased arterial stiffness, increased PP and systolic hypertension‐mediated target organ damage.^19^, ^20^, ^22^, ^23^, ^24^ In our middle‐aged patients, increased arterial stiffness might be partly explained by the higher prevalence of hypertension, metabolic syndrome and significantly greater waist circumference, which all are known risk factors of CV disease and mortality.^25^

Furthermore, age had a closer correlation with PWV, a marker of arteriosclerosis in younger patients, but with cIMT, a marker of atherosclerosis in middle‐aged patients, and this correlation was independent of hypertension and other established CV risk factors. Indeed, in a community dwelling cohort of men and women aged 20–101 years, it was shown that among individuals > 40 years, central arterial stiffening occurred independently of BP levels.^26^ Similarly, some previous reports have shown that most traditional CV risk factors measured at middle‐age lost their power during the aging process in terms of the association with incident CV disease.^8^ Particularly, systolic BP was among the individual risk factors showing significant interaction with age regarding incident ischemic stroke (*p* = .0067). Abbott and coworkers showed that the impact of BP and smoking declined by aging, but diabetes continued to be a risk factor at all ages.^9^ Olesen and coworkers demonstrated that age enhanced the association between 24‐h BP components and hypertension‐induced target organ damage through arterial stiffness, but did not affect association between 24‐h BP and atherosclerosis.^17^ Compared to clinic BP, 24‐h ambulatory BP has been shown to have a stronger association with hypertension‐mediated organ damage^10^ and poor outcome in stroke patients.^27^ The prognostic value of ambulatory BP components are also age‐dependent, diastolic BP being the best prognostic variable in younger individuals whereas PP in the elderly, reflecting different pathophysiological mechanisms of hypertension in younger and older hypertensive patients.^28^ Typically, our middle‐aged patients had significantly higher clinic PP and 24‐h PP, and an 8‐fold higher prevalence of increased cIMT (≥0.9 mm) as well as a 16‐fold higher prevalence of PWV > 10 m/s, both validated markers of subclinical target organ damage.^12^ Furthermore, in our multivariable‐adjusted models, 24‐h PP was associated with arterial stiffness and cIMT in middle‐aged patients, but not in the younger group. In a recent study of 4195 stroke patients (44% < 60 years), PP was significantly associated with long‐term stroke outcomes.^29^ Of note, this association was more pronounced in patients older than 60 years of age. However, 24‐h BP components, which have stronger prognostic value than clinic BP, were not part of their study. PP has also been shown as a stronger predictor of stroke than systolic and diastolic BP, even independent from systolic BP in middle‐aged patients, and its importance as CV risk factor increased with age.^30^

The changing pattern of BP with increasing age is widely known. The best predictor of CV disease shifts from diastolic BP to systolic BP and then to PP in parallel with increasing age, probably due to the fact that large artery stiffening and subsequent increase in PP is a phenomenon of higher age, while the resistance of peripheral small arteries (diastolic BP) is a dominant risk factor at younger age.^31^ Finally, PP has been identified as a potential determinant of several cardiac risk markers such as left atrium size, LV mass, cIMT and increased conduit artery stiffness,^32^, ^33^, ^34^ which all are well‐known predictors of stroke. The pathophysiological mechanism of why 24‐h diastolic, but not 24‐h systolic BP, was independently associated with vascular remodeling in stroke patients, should be explored in a larger prospective study in the future.

Current study has some limitations. This analysis was based upon cross‐sectional data. Whether increased arterial stiffness is the cause or a consequence of hypertension, and to what extent age modulates this relationship, should be explored in larger prospective longitudinal studies in future. However, Mendelian randomization (causal inference) analyses in recent genetic studies indicate a bi‐directional causal association between BP and arterial stiffness.^35^ Further, mainly Caucasians were included in this study, and the results should be cautiously interpreted for other ethnic groups. As previously reported,^7^ due to serious sequelae after the ischemic stroke, death, and other reasons for drop‐outs for a cardiac follow‐up, PWV was not measured in 32 patients and 24‐h ABPM was not examined in 64 patients. However, age and common clinical parameters did not differ significantly between those who had missing PWV and 24‐h ABPM data, and the patients included in the present study. Finally, ABMP and arterial stiffness indices were not measured at the same time point. However, it is known that BP is often labile at the early stages (weeks) following acute stroke, which may affect both PWV and 24‐h ABPM values.

## 5 CONCLUSIONS

Middle‐aged ischemic stroke patients had significantly higher burden of concomitant comorbidities and markedly increased arterial stiffness indices compared to younger patients. Age was strongly associated with arterial stiffness in younger patients, but its impact on arterial stiffness declined in middle‐aged patients. 24‐h pulse pressure was more closely related to arterial stiffness in middle‐aged patients. These results highlight the importance of hypertension, and the associated arterial damage in middle‐aged ischemic stroke survivors. Hence, every effort should be made to control cardiovascular risk factors, particularly hypertension, as early as possible in order to reduce the risk of premature arterial aging and early vascular events.

## CONFLICT OF INTEREST

There are no conflicts of interest for any authors.

## AUTHOR CONTRIBUTIONS

Sahrai Saeed, Ulrike Waje‐Andreassen and Peter M. Nilsson contributed to study conception and design and drafted the paper. Sahrai Saeed, Ulrike Waje‐Andreassen, Halvor Naess and Annette Fromm contributed to data acquisition. All authors contributed to the data interpretation, critically revised the paper and gave final approval.
